# A qualitative study of influences on the uptake of contraceptive services among people of reproductive age in Uganda

**DOI:** 10.1186/s12905-023-02274-7

**Published:** 2023-03-25

**Authors:** Cissie Namanda, Lynn Atuyambe, Sarah Ssali, Aggrey Mukose, Nazarius Mbona Tumwesigye, Frederick E. Makumbi, Ritah Tweheyo, Andrew Gidudu, Carole Sekimpi, Catherine Verde Hashim, Martha Nicholson, Peter Ddungu

**Affiliations:** 1 Makerere University School of Public Health New Mulago Hospital Complex, P.O. Box 7072, Mulago Hill Road, Kampala, Uganda; 2 Makerere University School of Gender and Women studies, P.O. Box 7062, Makerere Hill, Kampala, Uganda; 3 Marie Stopes Uganda, Plot 1020 Rose Lane, Kisugu-Muyenga, P.O Box 10431, Kampala, Uganda; 4 Marie Stopes International, 1 Conway Street, Fitzroy Square, W1T 6LP London, UK

**Keywords:** Influence, Modern contraceptive methods, Family planning

## Abstract

**Background:**

Uganda has registered an increased investment in family planning (FP) programs, which has contributed to improvement in knowledge of modern contraceptive methods being nearly universal. However, this has not matched the uptake of modern methods or the reduction in the unmet need for FP. This may be explained by the different influences which include health workers, family, and friends. Due to the limited uptake of contraceptive methods, a program on improving awareness, access to, and uptake of modern contraceptives is being implemented in selected regions in Uganda. We, therefore, conducted a formative study to determine the influences on contraceptive uptake at the onset of this program.

**Methods:**

Using a qualitative study design, we conducted thirty-two focus group discussions and twenty-one in-depth interviews involving men and women of reproductive age. We also carried out twenty-one key informant interviews with people involved in FP service delivery. Data was collected in four districts where implementation of the program was to take place. Audio recorders were used to collect data and tools were translated into local languages. A codebook was developed, and transcripts were coded in vivo using the computer software Atlas-ti version 7 before analysis. Ethical clearance was obtained from institutional review boards and informed consent was sought from all participants.

**Results:**

From the study, most married people mentioned health workers as their main influence while adolescents reported their peers and friends. Religious leaders and mothers-in-law were reported to mainly discourage people from taking up modern contraceptive methods. The cultural value attached to having many children influenced the contraceptive use decision among people in rural settings. Other influences included a person’s experience and housing.

**Conclusions:**

Health workers, religious leaders, and mothers determine the uptake of contraceptive services. The study recommends the consideration of the role of these influences in the design of FP program interventions as well as more involvement of health workers in sensitization of communities about contraceptive methods.

## Background

Uganda has one of Africa’s fastest-growing populations with an annual growth rate of 3% and a total fertility rate of 5.4 births per woman [1–3]. Uganda’s population is largely young, with nearly half being children 0–14 years, 20.6% aged 15–24 years, 28.6% aged 25–64 years, and only 2.7% of the population being 65 years or older [4]. Increased use of modern methods of contraceptives is an effective way of addressing the effects of unintended childbirth and the total fertility rate [5]. With increased investment in Family Planning (FP) programs [6], the country registered a decline of 27% in total fertility rate (TFR) over the last 28 years. The decline in TFR from 7.4 to 5.4 was mainly observed in the urban settings where an increase in contraceptive use and a reduction in maternal mortality ratio have also occurred [7, 8].

Knowledge of contraceptives in Uganda is nearly universal, according to the 2016 Demographic and Health Survey (UDHS), with 99% of both men and women having heard of at least one modern method of contraception [9]. However, factors such as median age at sexual debut and age at first marriage have been slow to change, especially in rural settings. Values for sexual and reproductive health indicators have not matched the set targets in Uganda’s FP costed implementation plan 2015–2020 [10]. Relatedly, modern contraceptive use among married women increased by only 9% between 2011 and 2016, with a significant unmet need of 28% [7].

The large variations and limited change in modern contraceptive indicators have been due to the different influences noted by different studies and reports [11–14]. Health workers are reported as one of the greatest influences on contraceptive use. Studies from different parts of Africa have noted this category to have an impact on people’s use of modern methods since they are responsible to disseminate health information to clients who visit health facilities [12, 15, 16]. Religion is another influence that has been reported and varies by faith. Whereas the Roman Catholics strongly preach against use, Anglicans advocate for contraceptive use while Moslems are divided on the issue [17, 18]. However, a study from Tanzania found that irrespective of people’s faith, they still used these methods due to their benefits and challenges for large families [19]. The behavior of people’s friends regarding contraception use is another factor that greatly influences the use. Friends and family often behave similarly. It has been found that users often influence their friends and family to also use these modern methods [20]. Given this information, the paper presents findings of influences on contraceptive uptake assessed at the start of the program.

Marie Stopes International (MSI) on behalf of the Ministry of Health (MoH), is leading a consortium of Family Health International FHI360, Population Media Centre (PMC), Reach A Hand Uganda (RAHU), and Makerere University School of Public Health (MakSPH) to implement a five-year program titled “Reducing High Fertility Rates and Improving Sexual Reproductive Health Outcomes in Uganda (RISE)”. The RISE program funded by UK Aid aims to increase awareness, access to, and uptake of high-quality FP services and strengthen the Public and Private sector capacity in FP service provision. This program was implemented in seven of the eleven UDHS 2011 regions in 75 districts. During the first year of implementation, a formative study was conducted with aim of determining key influences of contraceptive uptake in the program areas.

## Methods

### Design and study area

We used a qualitative study design utilizing a Formative Research approach to aid decision-making during the planning, design, and production of RISE SBCC intervention materials [21]. Focus group discussions (FGDs), Key informant interviews (KIIs), and in-depth interviews (IDIs) were conducted with participants of reproductive age as the primary intended beneficiaries of RISE SBCC.

Four of the seven regions were selected for this study to represent RISE implementation regions. The selection of the seven RISE regions was based on an earlier desk review that highlighted underserved regions or those with poor FP indicators. The regions are Karamoja, Eastern, East Central (currently divided into Bugisu, Bukede, and Teso), Central 1 (currently known as South Buganda), Central 2 (currently known as North Buganda), Western (currently divided into Bunyoro and Tooro), and West Nile. From these, the districts of Moroto in Karamoja, Mbale from Eastern (Bugisu) Mubende from Central 2, and Kibaale for Western (Bunyoro) were purposively selected. Mubende and Kibaale provided a semblance of rural districts while Mbale is a semi-urban district. Moroto represented areas that are remote with challenges of FP service delivery and unique cultural settings.

### Study methods, selection of study participants, and data collection

We conducted 32 FGDs with 259 participants from all the districts. Eight FGDs were conducted per district each with an average of eight people. The FGDs were disaggregated by age, sex, and marital status, from different sub-counties. We had four age categories that included 15–19, 20–24, 25–34, and 35+. We disaggregated all age groups by sex however for 15–19 and 20–24, we further categorized them into married and unmarried. We selected two categories from each age group making a total of eight groups per district. With guidance from the districts, we randomly selected two sub-counties per district. The participants were purposively selected using the interview group disaggregation criteria. Further participant selection was guided by the community leaders who were part of the community. Slightly more than half (54.8%) of the participants were women. 53% and 41% had completed primary or secondary respectively as their highest level of education. The median age was 23 years for both women and men. Nearly all had formal education either at the primary or secondary level with more than half being married.

In addition, 21 in-depth interviews with purposively selected women, stratified into those who were using any modern contraceptive at the time of the study (current contraceptive users), those who were not using (non-current contraceptive users), and those who had switched methods more than once (Switchers). From these categories, it was anticipated to generate information on why people either used, did not use, or had switched from one modern method to another. They represented categories of intended users required to explore their experience with contraceptive use. Half of the participants were contraceptive non-users while 8(33.3%) had switched methods of contraceptive. Nearly all were married and all had attained a primary level of education.

Twenty KIIs, 5 per district were conducted with district health officials (DHO), health workers (HWs)/service providers, village health team (VHT) members, community leaders, and Local council leaders (LCs). The sex of the KI interviewed was equally distributed. On average, women were older than men (50.7 vs. 43.2 years). District health team health workers and VHTs each contributed a fifth of KII participants and two-fifths were Local Councils leaders. Nearly all were married and had attained at least primary-level education. Details of all conducted interviews are seen in Table 1.

**Table 1:** Summary of all interviews conducted per district

Data collection method	FGDs	IDIs- women only	KIIs
Total Number	32	21	20
Number Per district	8	5	5
Participants’ summary	Disaggregated by age, sex, and marital status. Participants from at least 4 sub-counties.	Stratified into modern contraceptive current users, non-current users, and switchers.	District Health Team, Health workers, Village health teams, local council leaders, and or community leaders.

Interviews were mainly conducted in the local language or the language preferred by the respondent. Specific interview guides were written to guide each type of data collection. The tools were designed to explore themes related to the overall study objectives which included establishing the attitudes, barriers, key influences, key sources of information for contraceptive uptake, and media consumption habits in the project areas. All qualitative data were audio recorded and notes were taken. At the end of each field day, these data were backed up on the study computer to minimize risks of loss. We recruited 16 qualitative experienced research assistants who were determined based on the qualitative research experience and the commonly spoken language in the respective study areas. These were trained on concepts of the areas of the study, study objectives, procedures, tools, use of qualitative software Atlas TI Version 7, and research ethics and tools pretested. Research assistants worked with VHTs and Local community leaders to recruit community participants. Health workers involved in family planning provision were selected due to their expertise in working with contraceptive clients. Key informants included Village health teams and community leaders due to their involvement in health activities and mobilizing the community to receive services, district Health teams since they head health services in the districts, Secretary for women, and district health educators based on their involvement in family planning activities.

Informed consentwas sought from each study participant before we engaged them in participating. Adolescents aged 15-17years who were neither married, pregnant nor ever given birth, provided assent alongside parental/guardian consent. Adolescents who were pregnant or had given birth or married were treated as emancipated minors as per the national ethical guideline [22, 23].

### Data management and analysis

Audio recordings and notes were translated and transcribed. Audio files and transcripts were stored on a password-protected hard drive for safety. An initial codebook was developed after a debriefing meeting from a few transcripts of each category. The codes and transcripts were entered into a computer software Atlas-ti version 7 for further analysis. Two independent qualitative analysts coded the same transcripts using the codebook earlier developed. Coded transcripts from the two research teams were compared, and the text in the different coded transcripts was harmonized. Another independent analyst was available in case of emerging disagreements. Using thematic latent content analysis [24], we generated a matrix of code counts and made comparisons between the characteristics of the study participants as well as the methods. The codes, condensed sub-categories, and categories that emerged from the data form sections of the study findings described into priority themes. This paper presents findings on the theme of influences.

**Fig. 1:**
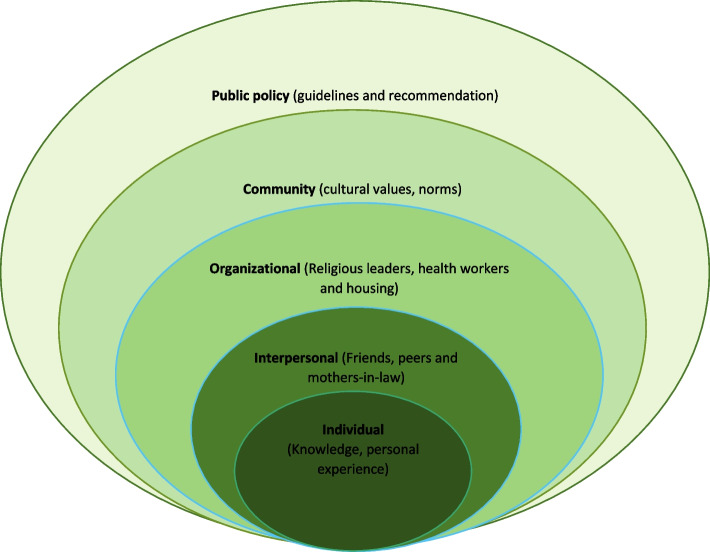
Study model as adapted from Bronfenbrenner socio-ecological model

The analysis was guided by a model as shown in Fig. 1, which was adapted from Bronfenbrenner’s socio-ecological model (SEM) [25]. This socio-ecological model postulates that human beings create environments in which they live and therefore to understand humans, we need to understand society as a whole and the changing environment in which they live. The model displays nesting circles with the individual at the centre followed by the interpersonal, organizational community then public policy.

The individual’s knowledge and skills help the person to understand more about contraceptive methods hence influencing their decision-making. The interpersonal level focuses on how people’s relationship with family and friends impacts decisions on contraceptive uptake. At the organizational level, a person’s decision relating to contraceptive method uptake will come from varying sectors like religion. In the model, the community level relates to cultural values and norms that affect the contraceptive methods status of a person. Lastly is public policy, the governing body in charge of family planning services. They develop guides and laws related to contraceptive uptake [25].

Therefore, from our study analysis, we explored these influences at the different levels of SEM as indicated in Fig. 1.

## Results

**Fig. 2:**
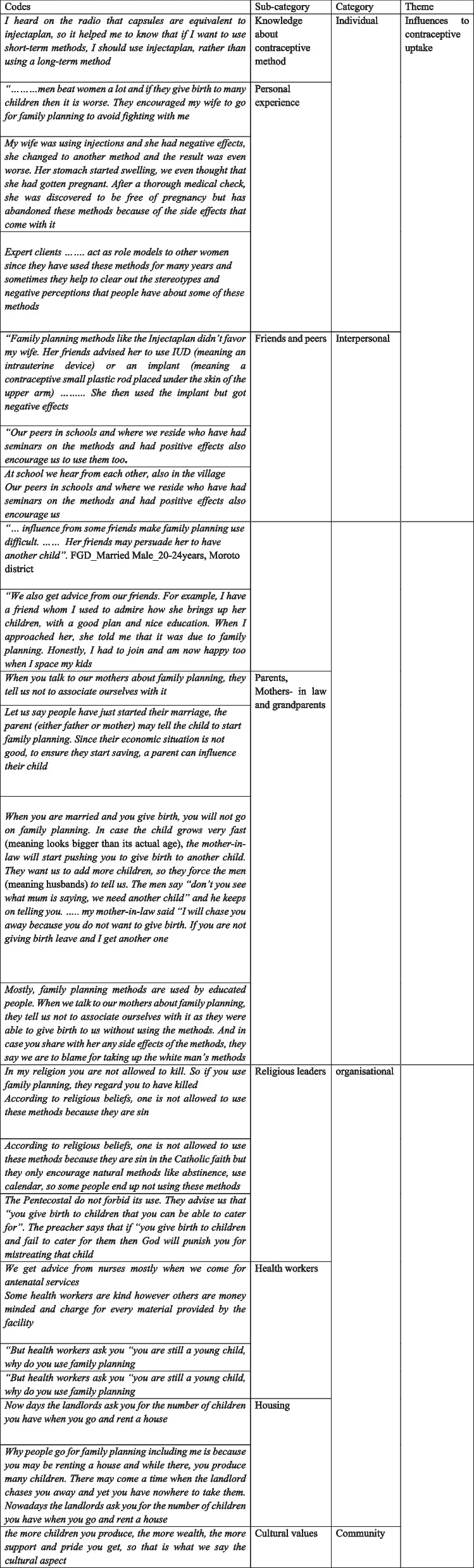
Summary of results

Our study results are presented in categories. The major categories that emerged from the theme of influences on contraceptive use were individual, interpersonal, organizational, and community as indicated in Fig. 2. These categories were developed from sub-categories that included knowledge, personal experiences, friends and peers, Mothers- in law and grandparents, religious leaders, health workers, housing, and cultural values.

### Influences on contraceptive uptake

#### Individual influences

##### Knowledge about contraceptive method

As seen from the first level of SEM, knowledge of contraceptive methods among people of reproductive age was reported to influence the uptake. This came mostly from the Kibaale and Moroto districts which are rural or have a scarcity of services respectively. A participant from the Kibaale district reported having switched to a short-term method due to the knowledge she had received about contraceptive methods. People involved in contraceptive service delivery also mentioned that the knowledge gap about contraceptive methods influences people not to seek these services.

*“I was using capsules, I heard on the radio that capsules are equivalent to injectaplan (meaning Depo-Provera contraceptive injection), so it helped me to know if I want to use short-term methods. I should use injectaplan, rather than using a long-term method. That’s why am using it.”* IDI-contraceptive Switcher, Kibaale district

##### Personal experience

Across all age categories, the personal experiences faced by participants influenced their use of contraceptive methods. In Kibaale male participants reported that women who had faced domestic violence from their spouses were forced to use contraceptive methods.

*“………men beat women a lot and if they give birth to many children then it is worse. They encouraged my wife to go for family planning to avoid fighting with me.”* FGD_Male_20-24years, Kibaale district

Experience of side effects was raised by male participants from almost all study districts as an influence for none use of modern contraceptive methods. The men whose spouses had faced this challenge from previous use were afraid of modern methods.

*“My wife was using injections and she had negative effects, she changed to another method and the result was even worse. Her stomach started swelling, we even thought that she had gotten pregnant. After a thorough medical check, she was discovered to be free of pregnancy but has abandoned these methods because of the side effects that come with it.”* FGD_ Maleale_35 + year, Mubende district

Nevertheless, positive experiences from expert clients who had used contraceptive methods for a long time acted as role models and cleared the negative perceptions.

*“Expert clients ……. act as role models to other women since they have used these methods for many years and sometimes they help to clear out the stereotypes and negative perceptions that people have about some of these methods.”* KII VHT Moroto.

#### Interpersonal influencers

##### Friends and peers

Friends and peers influenced the decisions on contraceptive methods uptake as unanimously reported during the 20 FGDs, six IDI, and six KII participants. Of the FGDs, six were adolescents, and the rest were of other ages. Discussions held with youth from all four districts proposed peers and friends having a big contribution to the decisions they made about contraceptive methods. Adolescent males stated that females of their age group were the category that was mostly influenced by peers and friends to use contraceptive methods. Adolescents from Kibaale who had used contraceptive methods encouraged their colleagues to use the same methods.

*“Family planning methods like the Injectaplan didn’t favor my wife. Her friends advised her to use IUD (meaning an intrauterine device) or an implant (meaning a contraceptive small plastic rod placed under the skin of the upper arm) ……… She then used the implant but got negative effects, ………”* FGD_Male_20-24years_Mubende district_.

*“Our peers in schools and where we reside who have had seminars on the methods and had positive effects also encourage us to use them too*.**”** FGD_ Female _15-19years, Mubende district.

Information about family planning was also shared among peers and friends. In Mubende which is predominately a rural district, adolescents shared information related to sexual issues while at school or in their places of residence. They acknowledged that people of their age lacked information on sexual health and got recommendations from their friends who had used the methods and not had side effects.

*“At school, we hear from each other, also in the village, we do talk to each other about health issues.”* FGD_Male_15-19years, Mubende district

In contrast, peers in the Moroto district advised each other that contraception was not good and that young people should give birth to many children like it is in their tradition.

*“… influence from some friends make family planning use difficult. …… Her friends may persuade her to have another child”.* FGD_Married Male_20-24years, Moroto district

Focus Group Discussions with older participants (20 years and above) emphasized that peers and friends particularly influenced contraceptive non-use when they had experienced negative effects. Across all districts, it was reported that friends of these age groups shared a lot of their experience and side effects faced from contraceptive use which information created fear among intended users.

*“Depending on what those that have used it say about how bad contraceptives make them feel, they have unending periods whereby every week they are in their periods. This is the reason I fear it”* FGD_Female_20-24years, Kibaale district

Despite the discouraging information from friends and peers of this age group, people in Moroto and Mubende who had friends that had successfully used modern contraceptive methods with healthier well-spaced, and educated children positively influenced colleagues to also utilize these methods. The friends acted as role models who would encourage families that have many children to go for contraceptive services as well as switch to alternative methods in case of undesired effects.

*“We also get advice from our friends. For example, I have a friend whom I used to admire how she brings up her children, with a good plan and nice education. When I approached her, she told me that it was due to family planning. Honestly, I had to join and am now happy too when I space my kids.”* FGD_Female_25-50years, Moroto district

From the IDIs, friends being influencers among users was only reported in the Kibaale district. Friends also advised families with many children but facing financial challenges, to use contraceptives. The influence corresponded to the type used, whether a person used it or not as reported by switchers and non-users. The potential contraceptive clients were mainly told about side effects which made them switch methods or total abandonment of use. This submission was further emphasized by KII respondents were friends who mainly shared information about the negative effects of modern contraceptives which caused their colleagues to shun their use.

##### Parents, mothers- in law and grandparents

Our data from FGDs and KIs showed that parents, mothers-in-law, and grandparents are influencers of contraceptive use in different ways. Our data from the 32 FGDs in the four districts pointed out that parents especially mothers mainly encouraged their children to use contraceptives. The parents gave this advice in circumstances where their children had husbands who were seen as uncaring, had no land to raise children (grandchildren,) and or with economic difficulties. This was noted mostly from Mbale which was an urban study district. In Moroto, it was reported that some mothers even take their married children to receive contraceptives method after the latter have had many children whereas users from Mubende were advised not to stop using family planning as a way to avoid suffering.

*“Let us say people have just started their marriage, the parent (either father or mother) may tell the child to start family planning. Since their economic situation is not good, to ensure they start saving, a parent can influence their child.”* FGD_Female _20_24years, Mbale district

In contrast, reports from rural study districts showed that some mothers discouraged their married or sexually active children from using contraceptives stating side effects, causing future fertility challenges, and advising that they instead produce like how their parents gave birth to them. Also, parents from small families discouraged their children to have few offspring as a way to widen the family.

Mothers-in-law and grandparents specifically advised their daughters’-in-law not to use modern contraceptive methods. In addition, our results show a few mothers-in-law were reported to even force their sons to tell their wives not to use. They mostly wanted the daughters-in-law to have many children for their sons which led some women to use contraceptives stealthily. In Kibaale, a discussion with women aged 20-24years echoed strongly about the threat they got from mothers-in-law some threatening to chase them away from the marriage if they did not want to give birth. Some in-laws even related modern contraceptives use to being promiscuous. Only a few FGD respondents reported a positive influence on contraceptive use from their in-laws due to the high cost of living.

*“When you are married and you give birth, you will not go on family planning. In case the child grows very fast* (meaning looks bigger than its actual age), *the mother-in-law will start pushing you to give birth to another child. They want us to add more children, so they force the men* (meaning husbands) *to tell us. The men say “don’t you see what mum is saying, we need another child” and he keeps on telling you. …. my mother-in-law said “I will chase you away because you do not want to give birth. If you are not giving birth leave and I get another one.’* FGD_Female _20-24years, Kibaale district.

From Mbale, a nonuser mentioned that some grandparents advised their granddaughters to quit contraceptive use due to side effects. They recommended the use of traditional family planning methods like safe days.

“*Mostly, family planning methods are used by educated people. When we talk to our mothers about family planning, they tell us not to associate ourselves with it as they were able to give birth to us without using the methods. And in case you share with her any side effects of the methods, they say we are to blame for taking up the white man’s methods*” FGD_Male_20-24years, Mubende district

#### Organizational influences

##### Religious leaders

As different religious sectors reached individuals, they took it upon themselves to either encourage or discourage contraceptive use. Both Christian and Muslim religious leaders were reported to negatively influence the use of modern contraceptive methods, especially in rural study districts across all age categories for both sexes. The majority of participants from FGDs mentioned that religious leaders advise people to continuously give birth as it’s written in the bible and to avoid depopulation.

It was further emphasized that faith-based beliefs related contraceptive use to intended abortion and regarded it as a sin. They preached to their congregation that those who used contraceptives will be punished by God. This message was preached mostly by Catholics, Moslems, some Pentecostals, some Anglicans, and other sects. There was a sect that operated in Kibaale and Mubende districts that had many followers and barred people from using contraceptives. Most preachers emphasized the myths and side effects of contraceptives as dangers faced by people who use these methods.

*“In my religion, you are not allowed to kill. So, if you use family planning, they regard you to have killed.”* FGD_Female_20-24years, Mubende district

Natural methods were the only recommended options and lessons on how to use natural methods were given during marriage counseling for those soon-to-wed.

*“According to religious beliefs, one is not allowed to use these methods because they are sin in the Catholic faith but they only encourage natural methods like abstinence, use calendar, so some people end up not using these methods”* FGD_ Female _20-24years, Moroto district.

However, male and female FGD participants from Mubende mentioned that a few Pentecostal leaders advised people to use modern contraception and give birth to children they could afford to care for. Similarly, in Kibaale a discussion with females of 20-24years stated that places of worship hosted people who sensitized the congregation about contraceptive use. In addition, a quarter of respondents from FGDs mostly in urban study areas acknowledged that people appreciate the value of using contraceptives compared to the expense of having so many children.

*“The Pentecostal do not forbid its use. They advise us that “you give birth to children that you can be able to cater for”. The preacher says that if “you give birth to children and fail to cater for them then God will punish you for mistreating that child”.”* FGD_ Female_35 + years, Mubende district.

Across the IDIs and KIIs, participants equally held this view of religious leaders against modern contraceptives with the one exception of a contraceptive user who mentioned that a Catholic priest had at one time encouraged them to uptake family planning methods.

“*On Easter, a priest told us that you’re overproducing, use family planning and educate your children*.” IDI contraceptives user, Kibaale district

##### Health workers

From all interviews and study areas, health workers were reported as influences on people’s uptake of contraceptives. However, there was a difference in how they influenced the different age groups. Age groups of 20-34years for men and women reported health workers as positive influences since they offered counselling and advice at health facilities and communities. The counselling was often given to women who came for antenatal, postnatal, and immunization services especially those with many children. Health workers also moved into communities to sensitize on contraceptives, provide outreach services, and participated in radio talk shows for health education as a means of sensitizing the public on the available methods and encouraging use.

“*We get advice from nurses mostly when we come for antenatal services. They tell us that we have to use family planning methods available in the hospital after giving birth*.” FGD_ Female _25-50years, Moroto district

Same reports were given by teenage mothers in the Moroto district, with teenage and young fathers reporting to have been counselled and asked to go home and talk to their spouses to use contraceptives and space their children.

In contrast, health workers discouraged unmarried adolescents, those not in a union, or those who had never given birth from using contraceptives. Adolescents from Mbale and Mubende districts reported being denied contraceptives by health workers on account of being young. Other providers asked for money which the young people did not have since the majority are not employed or lacked any source of income. Some were reported to be rude to these adolescents.

*“But health workers ask you “you are still a young child, why do you use family planning?”* FGD_Female_20-24years, Mbale district

*“Yes. Some health workers are kind however others are money minded and charge for every material provided by the facility. So, we know these workers by face and shun them away when we find them at the facility”* FGD_Female_15-19years, Mubende district

In Mbale, adolescents further revealed feeling stigmatized when they were asked by health workers why, they were engaging in sexual relationships at a young age. They were also asked to be escorted to the health facilities by their parents before providing them with contraceptives.

*“What makes girls like us fail to go for family planning is that when you go to the health centre the health workers say “eeeh, you have already started sexual relations with men? Go home”. So, you do not go back again.”* FGD_Female_15-19years, Mubende district.

*“……. if you go with your mum or auntie they will work on you but if you go alone, health workers will refuse. The health workers will tell you to go back and come back with a parent.”* FGD_Female_20-24years, Mbale district.

Participants from IDIs emphasized the role of health workers in contraceptive method uptake. It was mentioned that persons who sought information from health workers after being discouraged by their friends were able to take up contraception. They reported having received counselling on side effects and options of different methods.

*“I often hear women mostly the old ones, discussing that FP is bad. That if you start using it, you might develop some sickness and even die. They say that whether they produce ten or twenty children, you cannot see them start to use FP methods. It makes me feel bad about starting to use FP methods yet I would have loved to start using them. …… However, health workers have helped me. When health workers tell you something you also trust in it. I never trusted in family planning, but now I have started using it”* IDI User, Mubende district.

Health workers also reportedly routinely urged male spouses to encourage their wives to turn up for family planning services.

*“Health workers do influence a lot to use the family planning method. “You just need to talk to your wife and tell her what you heard from the health worker. “I always go home and share with her the importance of family planning, she will listen and accept it”* FGD_Male_15-19years, Moroto district

However, an individual who wasn’t using contraceptives in Kibaale, reported that health workers who were not well trained in FP were not able to give comprehensive guidance and biased clients on the contraceptive methods to be used.

*‘There’s one health worker I inquired from, she/he wasn’t that much qualified. He told me that all family planning methods are bad. That the good one is the condom. But with condoms, you also know men, it reaches a time and they refuse.”* IDI_Contraceptive Nonuser, Kibaale District.

Village health team members influenced the community members to take up contraceptive methods as they provide information on the contraceptive methods and even supplied some like condoms. They were reported to be highly influential because they move door-to-door and respondents felt gave a personal touch and encouraged communities to ask openly about contraceptives. However, VHTs were reported to have limited knowledge of some contraceptive methods and thus could only offer narrow information.

*“The VHTs also help us, they do this on a one-on-one when you visit them. They will give you a range of options that you can use. Condoms, injector plan, and others but on an individual basis.”* FGD_ Male_15–19, Mubende district

##### Housing

The sector of housing influenced people’s decisions on contraceptive use. In the Mbale district which was more urban, challenges with housing influenced women’s use of family planning to reduce family size. Focus group participants reported that many property owners were not willing to rent their houses for accommodation to people with many children. Hence the people not owning houses were forced to use contraceptives so that they don’t get chased away from the properties they rented.

*“Why people go for family planning including me is because you may be renting a house and while there, you produce many children. There may come a time when the landlord chases you away and yet you have nowhere to take them. Nowadays the landlords ask you for the number of children you have when you go and rent a house”.* FGD_Female_25–35, Mbale district

#### Community influences

Culture as indicated in SEM was an influence reported within the Karimojong community. The importance of many children to this community surpassed the decision to use contraceptive methods as reported by male FGD participants across all age groups from Moroto district. It was mentioned that cultural leaders have a strong voice within the community where they emphasize the need of many children thus discouraging the use of contraception. In addition, our results showed that many children were equated to wealth and pride., The young people were asked to give birth and replace the old generation.

*“Long ago, in the years of our grandfathers and grandmothers* ***…*** *the order of the day was to produce children, the more children you produce, the more wealth, the more support and pride you get, so that is what we say the cultural aspect.* ***…*** *it is the elders”*. FGD_ Male _35-49years, Moroto district

The cultural practice of paying dowry for marriage encouraged communities to disregard contraceptives as the women aimed to give birth to many children. The children were to show appreciation to the husband and pay back the dowry.

***“*** *In Karamoja, once you have finished paying the dowry for the woman, it is not allowed for you to space children. The wife has to show appreciation by giving birth to children as long as the man wants, as a way to pay for all the cows he spent on the wife”* FGD _Male_15–19years, Moroto District.

*“Long ago, in the years of our grandfathers and grandmothers* ***…*** *the order of the day was to produce children, the more children you produce, the more wealth, the more support and pride you get, so that is what we say the cultural aspect.* ***…*** *it is the elders”*. FGD_ Male _35-49years, Moroto district.

Further, parents wanted their families to follow their lineage hence fathers ask their sons to have 3–4 wives and produce many children.

## Discussion

The Our study found several influences at the different levels of SEM that lead to, or discourage people from using contraceptive methods. The most reported positive influencers among the married or those in union were health workers. Mothers-in-law, grandparents, and, religious leaders were the main negative influencers. Friends and peers were mentioned especially among young people. Other influences included people’s knowledge and experience with contraceptive methods either influenced negatively or positively use. No influence was noted from the public policy level of SEM.

The finding of health workers being the major influence is not different from what was reported by another study in Uganda where men who had discussed modern contraceptive methods with health workers were found to use these methods [26]. This may be explained by findings from Nigeria and Ghana that reported health workers as the commonest source of family planning information [12, 15]. Relatedly, their positioning as healthcare givers especially among women of reproductive age during antenatal, postnatal, and immunization, allows them to sensitize about family planning as part of sexual and reproductive health information as recommended by WHO [11, 16]. Hence this increases the opportunities for health workers to improve the uptake of contraceptive methods given the trust people have in them.

Though health workers are held in a high position, unmarried adolescents indicated some of these service providers to stigmatize them with questions about why they are having sex and asking them to be accompanied by an adult for permission to receive services. Furthermore, they were denied information and asked for user fees for these services yet they were supposed to be accessed at no cost. Earlier studies conducted among this age group found cost as a limitation to the use of modern contraceptive methods as well as different forms of stigma from health workers [27, 28]. This implies that the country will experience an increase in teenage pregnancy, unsafe abortion, and adolescent maternal mortality if this age group is denied the free contraceptive services. Furthermore, to ensure improvement in contraceptive use, strategies to reach this age group should differ from those of adults.

Our study findings indicate friends and peers are a major influence among young people, not only health workers. This is an unusual finding that implies revised strategies to advocate and improve use for this age group. There is a need to involve young people who are already using modern contraceptive methods in the design of FP messages and actual method provision. Another surprising finding was on the housing where people who were renting houses to stay in used FP methods since owners did not rent out their houses to people with many children.

Both male and female participants mainly from rural settings agreed to religion mostly discourages people to use modern contraceptive methods. This finding is congruent with other studies conducted in different parts of Africa where the Moslem and Christian faith encourage the congregations to avoid the involvement in the use of artificial methods of controlling the number of children a couple brings into the world [19, 29, 30]. However, it’s also important to acknowledge that even though the spiritual worship messages are negative towards contraceptive use, urban residents listen to the preaching but go ahead and use these methods since they appreciate the benefits. In addition, the ability to provide for the children and existing financial constraints are other justifications for people to use modern contraceptive methods. Similarly, qualitative studies conducted in Tanzania and Nigeria found that ability to take care and provide for the children by those who brought them into this world was justification for the use of family planning methods [19, 31]. Therefore, it’s urged that irrespective of the couple’s faith beliefs, adequate reproductive health information should be given for informed choice on family planning use [31].

Though mothers especially from urban settings encouraged their daughters to use family planning methods due to economic challenges and bad marriages evidenced by gender-based violence, mothers-in-law and grandparents were more inclined to traditional customs and norms of having many children. In addition, culture was a strong influence verbalized in the Moroto district which is located in the poorest region of the country. It was echoed that a woman’s role was only giving birth and those that resorted to using these methods are often viewed as promiscuous. This finding is not different from what was reported in Ghana where respondents justified the bias of culture as a negative influence on not using modern methods of family planning [15]. Due to the power the mothers’ have over their daughters-in-law, the latter are forced to continue having children as a way to ensure harmony in family marital relationships.

Though the study did not have any influence at the public policy level of SEM, this has been reported to affect contraceptive use [32]. Sexual and reproductive policy components emphasize the provision of contraceptive services as a way to improve the uptake. In Uganda, the policy addresses these concerns and ensures the provision of these services at different health facility levels. The existence of such policy components influences the attention and availability of these services at health facilities whether public or private [33]. However, the adolescent health Policy is silent on comprehensive contraceptive services for this age group. This may explain the finding from our study where health workers did not provide contraceptive services to adolescents[34].

The strengths of our study are that it provides a good representation of different areas of the country since data was collected from rural, urban, and remote underserved settings. We also had a good representation of the population and marital status concerning contraception. The study was limited by accessibility due to participants in rural areas due to long travel and challenges with transport.

## Conclusion

Health workers were the major influences, especially among married people or those in a union. Majority of the religious leaders are negative influences of contraceptive methods. Adolescent users encouraged their colleagues who were sexually active to uptake contraceptives. Older people mainly shared negative information that discouraged their peers and friends from taking up these modern methods. Mothers-in-law and grandparents majorly wanted their children not to consider contraceptives. The knowledge people had in their personal experiences about contraceptives was another influence.

We recommend program implementers identify and work with religious leaders who are positive about the uptake of modern methods. These can act as change agents for their colleagues. In addition, SBCC messages should be designed in such a way that they encourage people to seek contraceptive information from qualified health workers as a way to reduce misinformation from peers and friends. Supporting satisfied users by equipping them with correct contraceptive information so that they can conduct peer-to-peer counseling. Furthermore, Community outreach activities should emphasize changing people’s myths about contraceptive methods. Health workers and VHTs with support from programmers can conduct FP outreaches in communities with limited information access as a way to increase access to correct health information. Inter personnel communication model may be a good strategy where VHTs involved in FP activities can have face-to-face interaction with dissatisfied users to address their concerns since these clients share a lot of negative experiences with their peers and friends. Implementers of family planning activities should pay attention to the training of health workers and VHTs as well as changing their attitude towards the provision of contraceptive methods to sexually active adolescents.

## Data Availability

The data that support the findings of this study are available from the corresponding author, who is the Study Coordinator, Makerere University School of Public Health, RISE Project, upon reasonable request.

## Abbreviations

DHO: District Health Officials
DFID: Department for International Development
FGDs: Focus group discussions
FP: Family Planning
HWs: health workers
IDIs: In-depth interviews
KIIs: Key informant interviews
LCs: Local council
mCPR: Modern Contraceptive Prevalence Rate
MMR: Maternal Mortality Ratio
MoH: Ministry of Health
RISE: Reducing High Fertility rates and Improving Sexual Reproductive Health Outcomes
SBCC: Social Behavioural Communication Change
SEM: Socio-ecological model
TFR: Total Fertility Rate
UBOS: Uganda Bureau of Statistics
UDHS: Uganda Demographic Health Survey
UNCST: Uganda National Council for Science and Technology
VHT: Village Health Team
